# Expression of IL-18, IL-18 Binding Protein, and IL-18 Receptor by Normal and Cancerous Human Ovarian Tissues: Possible Implication of IL-18 in the Pathogenesis of Ovarian Carcinoma

**DOI:** 10.1155/2014/914954

**Published:** 2014-05-15

**Authors:** Liat Medina, Alex Rabinovich, Benjamin Piura, Victor Dyomin, Ruthy Shaco Levy, Mahmoud Huleihel

**Affiliations:** ^1^The Shraga Segal Department of Microbiology, Immunology and Genetics, Be'er Sheva, Israel; ^2^Faculty of Health Sciences, Ben-Gurion University of the Negev, 84105 Be'er Sheva, Israel; ^3^Unit of Gynecologic Oncology, Division of Obstetrics and Gynecology, Soroka University Medical Center, 84105 Be'er Sheva, Israel; ^4^Institute of Pathology, Soroka University Medical Center, 84105 Be'er Sheva, Israel

## Abstract

Proinflammatory cytokine IL-18 has been shown to be elevated in the sera of ovarian carcinoma patients. The aim of the study was to examine the levels and cellular origin of IL-18, IL-18 binding protein, and IL-18 receptor in normal and cancerous ovarian tissues. Ovarian tissue samples were examined by immunohistochemical staining for IL-18, IL-18BP, and IL-18R and mRNA of these cytokines was analyzed with semiquantitative PT-PCR. IL-18 levels were significantly higher in cancerous ovarian tissues (*P* = 0.0007), IL-18BP levels were significantly higher in normal ovarian tissues (*P* = 0.04), and the ratio of IL-18/IL-18BP was significantly higher in cancerous ovarian tissues (*P* = 0.036). Cancerous ovarian tissues expressed significantly higher IL-18 mRNA levels (*P* = 0.025), while there was no difference in the expression of IL-18BP mRNA and IL-18R mRNA between cancerous and normal ovarian tissues. IL-18 and IL-18BP were expressed dominantly in the epithelial cells of both cancerous and normal ovarian tissues, while IL-18R was expressed dominantly in the epithelial cells of cancerous ovarian tissues but expressed similarly in the epithelial and stromal cells of normal cancerous tissues. This study indicates a possible role of IL-18, IL-18BP, and IL-18R in the pathogenesis of epithelial ovarian carcinoma.

## 1. Introduction

Epithelial ovarian carcinoma (EOC) is the most frequent cause of death from gynecologic malignancies and the fifth leading cause of death from all cancers in women [1]. The cytokine network in the tumor microenvironment may be involved in many aspects of tumor growth and spread such as proliferation, motility, survival, cell-cell or cell-matrix adhesion, neovascularization, extracellular matrix remodeling, host cell infiltration, and local immune response [2]. In previous studies, we have demonstrated that cancerous ovarian tissues (COT) express and secrete higher levels of IL-1*α*, IL-1*β*, IL-6, and TNF-*α* compared to normal ovarian tissues (NOT) and suggested that these cytokines may have a role in the pathogenesis of EOC [3–8].

IL-18, formerly known as interferon-*γ* inducing factor [9], is a pleiotropic, proinflammatory cytokine with dual effects on tumor development and progression [10]. On the one hand, IL-18 induces T helper type 1 (Th1) immune response, which is generally regarded as the immune reaction that acts against malignant tumors. On the other hand, IL-18 promotes T helper type 2 (Th2) immune responses that may inhibit recognition of cancer cells by immune cells, increase the adhesion molecules, induce production of angiogenic factors, and promote a prometastatic microenvironment [11, 12]. IL-18 belongs to the IL-1 family of ligands [13]; it has 12% homology with IL-1*α* and 19% homology with IL-1*β*. Similar to IL-1*β*, IL-18 is synthesized as an inactive precursor protein of 24 kDa (pro-IL-18). Pro-IL-18 is processed to a mature and bioactive molecule of 18.3 kDa by the intracellular cysteine proteinase caspase-1 (IL-1*β* converting enzyme—ICE) [14]. Additional proteases can process pro-IL-18 to both a biologically active form of the cytokine (caspase-4) and an inactive molecule of 14 kDa (caspase-3) [11]. Both mature and inactive forms of IL-18 can be secreted by cells.

IL-18 receptor (IL-18R) is a member of the IL-1 receptor family. It consists of a ligand-binding domain (*α*-chain), which binds IL-18 with low affinity, and a signal-transducing domain (*β*-chain) [15]. Together, a formed high-affinity IL-18R complex transduces its signal to stimulate the MAPK pathway [16].

IL-18 binding protein (IL-18BP) is a secreted 40 kDa glycoprotein which possesses a high affinity to IL-18. This constitutively expressed soluble protein prevents the binding of mature IL-18 to its receptor and inhibits IL-18 biological activity. Four isoforms exist in humans, displaying differential ability to bind and inhibit IL-18 activity [17].

Since its original description, IL-18 has been shown to be secreted by multiple cell types, including T and B cells, monocytes, macrophages, and some human tumors [18]. It is still unclear; however, which cell population is responsible for the elevated IL-18 levels in the sera of ovarian cancer patients and how IL-18 influences the development and progression of EOC [19]. The aim of the present study was to evaluate the expression levels and the cellular origin of the IL-18 family members (IL-18, IL-18BP, and IL-18R*α*) in NOT and COT.

## 2. Materials and Methods

### 2.1. Reagents

Phosphate buffered saline (PBS), Dulbecco's Modified Eagle's Medium (DMEM), fetal calf serum (FCS), L-glutamine, antibiotics combination of streptomycin and penicillin, and Trypsin-EDTA were all purchased from Biological Industries (Beit Haemek, Israel). BSA and Tween-20 were purchased from ICN Biomedicals, Inc. (Aurora, OH, USA).

### 2.2. Antibodies

The ELISA module set kit for human IL-18 (cat number BMS267MST) was purchased from Bender MedSystems (San Bruno, CA, USA). The ELISA DouSet kit for IL-18BP (cat number DY119) was purchased from R&D Systems, Inc. (Minneapolis, MN, USA). The monoclonal mouse anti-human IL-18 (cat number DY119, MBL, Naka-ku, Nagoya, Japan), the polyclonal goat anti-human IL-18BP (cat number sc-9461, Santa Cruz Biotechnology, Inc., Santa Cruz, CA, USA), and the monoclonal mouse anti-human IL-18R*α* (cat number MAB840, R&D) were used for immunohistochemical staining.

### 2.3. Origin and Handling of Ovarian Tissues

Fresh ovarian tissues were collected under sterile conditions from the operating room of the Department of Obstetrics and Gynecology, Soroka Medical Center, Be'er Sheva, Israel. Institutional review board approved the conduction of this study before its initiation. Ovarian tissue samples were obtained from 25 women having surgery for EOC (17 serous papillary adenocarcinoma, 3 endometrioid, 3 undifferentiated, and 2 clear cell carcinoma) (the vast majority of EOCs were stages III and IV) and 38 women undergoing surgery for benign (e.g., fibroid uterus) or malignant (e.g., endometrial hyperplasia or carcinoma) gynecologic disease other than ovarian carcinoma. The histopathologic diagnosis was confirmed in formalin-fixed, paraffin-embedded tissues. Fresh tissue samples were washed immediately with cold PBS in order to eliminate residual blood cells. Part of the tissues was stored at −70°C to be used later for the evaluation of proteins and mRNA levels and the other part was fixed in formalin for immunohistochemical staining.

### 2.4. Immunohistochemical Staining

Immunoperoxidase assay was carried out on paraffin-embedded NOT and COT sections using the Vectastain Elite ABC kit (Vector Laboratories, Burlingame, CA, USA). Briefly, 4 micron thick sections from formalin-fixed, paraffin-embedded tissue blocks were mounted on Superfrost Plus slides, dried at 37°C for 48 hours, and stored at room temperature. Before the primary antibodies were applied, the slides were deparaffinized in xylene, rehydrated in graded alcohol, and warmed twice in 6 M UREA for 5 minutes. Blocking of the nonspecific background staining was achieved with PBS containing 2.5% of either goat or rabbit serum. This solution was also used to dilute the primary antibodies: mouse anti-human IL-18, mouse anti-human IL-18R, and goat anti-human IL-18BP. The biotinylated antibody and the streptavidin-peroxidase conjugate (Avidin-Biotin Complex (ABC)) were applied according to the supplier's instructions (Vector Laboratories). Blocking of the endogenous peroxidase was done with 0.01% H_2_O_2_ in 80% methanol for 25 minutes before the ABC was applied. Development was done with 3,3′diaminobenzidine (DAB) and Mayer's haematoxylin was used for counterstaining. For negative control we used the blocking solution instead of primary antibodies.

### 2.5. Preparation of Normal and Cancerous Ovarian Homogenates

NOT and COT were homogenized in 1 ml cold saline in ice. At the end of the homogenization process, the mixture was centrifuged at 13000 RPM for 15 minutes; the supernatant was collected and stored at −70°C. Total protein was examined by Bio-Rad reagent (BIO-RAD, Hercules, CA, USA). IL-18 and IL-18BP levels were examined using specific ELISA kits.

### 2.6. Extraction of Total RNA and Semiquantitative RT-PCR Analysis

Total RNA was extracted from ovarian tissues using the EZ-RNA Reagent protocol (Biological Industries) according to the manufacturer's instructions. First-strand complementary DNAs (cDNAs) were synthesized from 2.5 *μ*g total RNA with 5× RT buffer, 2 *μ*M oligo (dT) primers (Sigma), 0.5 mM dNTP mix (ORNAT, Rehovot, Israel), 10 U Rnase Out (Invitrogen, Carlsbad, CA, USA), and 200 U M-MLV (Invitrogen) in a final volume of 20 *μ*L. The reverse transcriptase (RT) reaction was performed for 1 h at 37°C and stopped for 10 min at 65°C. The volume of 20 *μ*L was subsequently filled up to 60 *μ*L with DEPC (Sigma) treated water. Negative controls for the reverse transcriptase reaction (RT) contained DEPC treated water instead of RNA. The semiquantitative RT-PCR was performed by calculating the ratio between the intensity of each band (obtained by densitometry, using TINA 2.0 software) and the intensity of the *β*-actin band of the same cDNA sample. In brief, 2.5 *μ*L of cDNA was amplified by PCR in a final volume of 25 *μ*L containing 10× PCR buffer, 0.2 mM dNTP mix, 2 mM Mg^++^, 0.25 U DNA polymerase (BIOLINE, London, UK), and 0.5 *μ*M of the following primers: forward-5′ gacgaggcccagagcaagag 3′, reverse-5′ gggccggactcgtcatactc 3′ for the human *β*-actin (935 bp); forward-5′ caaggaaatcggcctctatt 3′, reverse-5′ tcctgggacacttctctgaa 3′ for hIL-18 (255 bp); forward-5′ cctggagtgaacagtccctga 3′, reverse-5′ aaccaggcttgagcgttcc 3′ for hIL-18BP (402 bp); and forward-5′ cagttgagttgaatgacacagg 3′, reverse-5′ tccactgcaacatggttaag 3′ for hIL-18R (424 bp). Negative controls for the polymerase chain reaction contained DEPC treated water instead of cDNA (cDNA-). The PCR reactions were carried out on a T personal Thermal Cycler (Biometra, Göttingen, Germany). All of the tested factors (cytokines and *β*-actin) were calibrated using several cDNA concentrations to determine the number of cycles needed for an appropriate amplification. The *β*-actin cDNA was amplified at 63°C for 30 cycles, the IL-18 was amplified at 57°C for 35 cycles, the IL-18BP was amplified at 62°C for 30 cycles, and the IL-18R was amplified at 57°C for 35 cycles. Twenty microliters of each PCR product were run on 2% agarose gel, containing ethidium bromide, and photographed under UV light.

### 2.7. Statistical Analysis

Samples were examined in triplicates in each experiment. Each experiment was repeated at least three times. Results are expressed as the mean ± SEM.

To evaluate statistical significance of the results, Student's *t*-test was performed; *P* value <0.05 was considered as significant.

## 3. Results

### 3.1. IL-18 and IL-18BP Protein Levels in NOT and COT Homogenates

**Figure 1:**
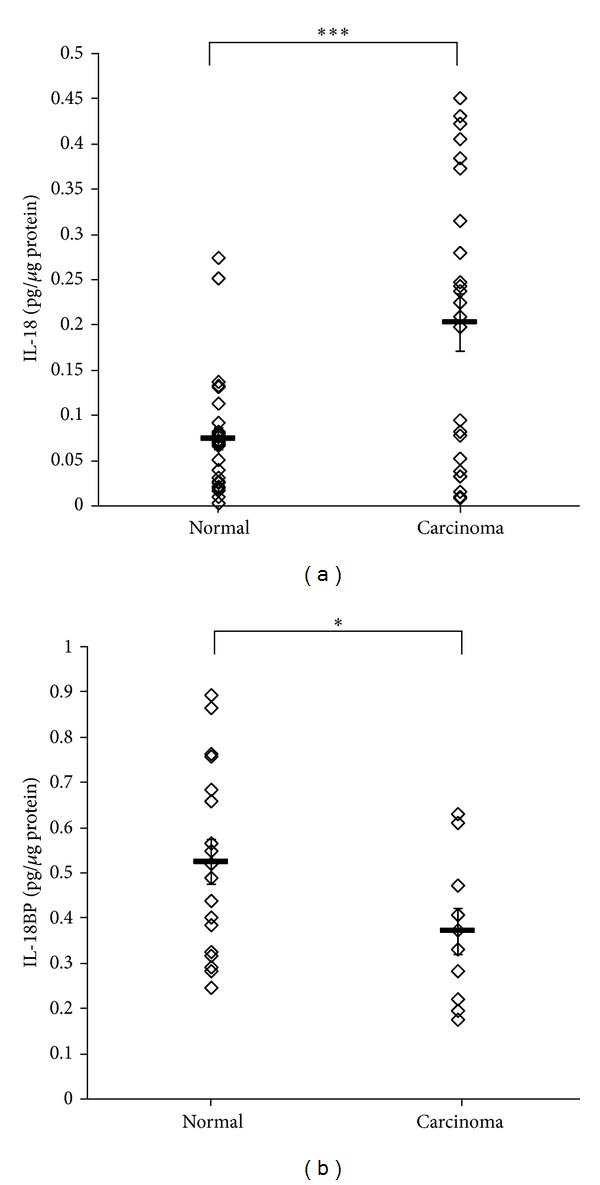
IL-18 and IL-18BP protein levels in normal and cancerous ovarian tissue homogenates. Normal and cancerous ovarian samples were homogenized. IL-18 (a) and IL-18BP (b) protein levels were evaluated by specific ELISA kits. Levels are expressed as pg/*μ*g protein; each point represents one of the positive samples; *n* = 28 (normal) and 24 (carcinoma) in (a) and 18 (normal) and 10 (carcinoma) in (b). Horizontal lines indicate MEAN ± SEM. ∗/∗∗∗ indicates statistical significance according to *t*-test: *P = 0.04; ***P = 0.0007.

Percentage of positive IL-18 expression was higher in COT homogenates compared to NOT homogenates: 96% and 73.7%, respectively (Table 1). Furthermore, the mean value of IL-18 levels expressed by positive COT samples was significantly higher compared to positive NOT samples: 0.2 ± 0.03 pg/*μ*g protein and 0.07 ± 0.01 pg/*μ*g protein, respectively (*P* = 0.0007) (Figure 1(a)). All ovarian homogenates samples examined, normal and cancerous, were found to have a positive expression of IL-18BP (Table 1). The mean value of IL-18BP expressed by the NOT was significantly higher compared to COT samples: 0.52 ± 0.04 pg/*μ*g protein and 0.37 ± 0.05 pg/*μ*g protein, respectively (*P* = 0.04) (Figure 1(b)). Since protein activity can be presented as the ratio between the expression levels of the protein and its inhibitor, we compared the IL-18/IL-18BP expression levels in 9 NOT samples and 10 COT samples. The mean value of IL-18/IL-18BP expression levels of the positive COT samples was significantly higher compared to NOT samples: 0.6 ± 0.17 and 0.14 ± 0.07, respectively (*P* = 0.036).

### 3.2. IL-18 mRNA, IL-18BP mRNA, and IL-18R mRNA Levels in Normal and Cancerous Ovarian Tissue Homogenates

The percentage of samples positive for IL-18 mRNA and IL-18R mRNA was higher in NOT compared to COT: 81.2% and 66.6%; 76.9% and 63.6%, respectively (Table 2). However, the percentage of samples positive for IL-18BP mRNA was higher in COT compared to NOT: 100% and 85.7%, respectively. As with IL-18 protein and IL-18R protein expression (Table 1), some of the ovarian homogenates, both normal and cancerous samples, did not express detectable levels of IL-18 mRNA or IL-18R mRNA (Table 2).

**Figure 2:**
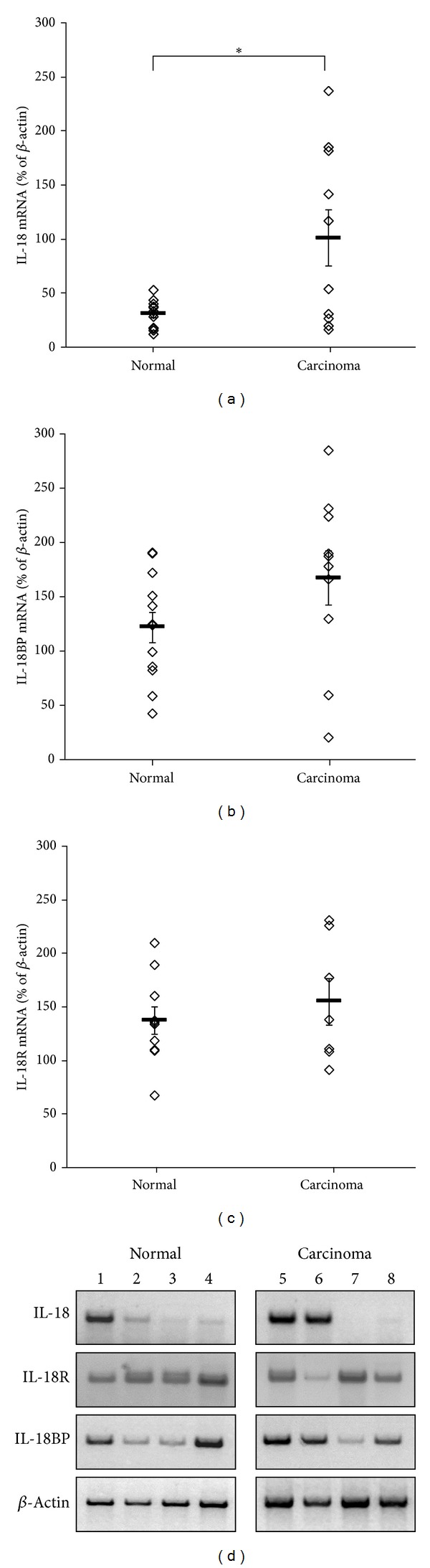
IL-18, IL-18BP, and IL-18R mRNA levels in normal and cancerous ovarian homogenates. Normal and cancerous ovarian samples were homogenized. The expression levels of IL-18 (a), IL-18BP (b), and IL-18R (c) mRNA were evaluated by semiquantitative RT-PCR. Quantitative evaluation of the positive PCR products was performed by densitometry. Each point represents the ratio between IL-18, IL-18BP, or IL-18R bands and *β*-actin bands of the same sample; *n* = 13 and 10 in (a), 12 and 10 in (b), and 10 and 7 in (c), for the normal and carcinoma samples, respectively. Horizontal lines indicate MEAN ± SEM. (d) Representative samples of normal (1–4) and cancerous (5–8) ovarian tissues are presented. ∗ indicates statistical significance according to *t*-test: *P = 0.025.

In positive tissue homogenates for IL-18 mRNA, COT expressed significantly higher IL-18 mRNA levels compared to NOT, as evaluated by RT-PCR and quantified by densitometry, with mean values of 100.8 ± 25.9% and 30.8 ± 3.43%, respectively (*P* = 0.025) (Figure 2(a)).

In positive tissue homogenates for IL-18BP mRNA, the expression levels of IL-18BP mRNA were slightly, but not significantly, higher in COT samples compared to NOT samples: 167 ± 25% compared to 121.7 ± 14.3%, respectively (Figure 2(b)). The IL-18R mRNA expression levels were similar in the COT homogenates (154.7 ± 21.7%) and in the NOT homogenates (137 ± 13%) (Figure 2(c)). mRNA expression patterns of IL-18, IL-18BP, and IL-18R as examined by RT-PCR are presented in Figure 2(d); four different samples of NOT (1–4) and COT (5–8) are displayed.

### 3.3. Cellular Origin of IL-18, IL-18R, and IL-18BP in Normal and Cancerous Ovarian Tissues

**Figure 3:**
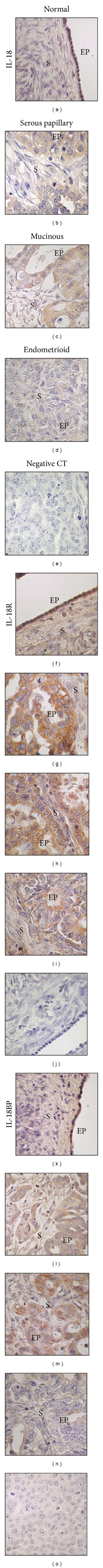
Immunohistochemical staining of normal and cancerous ovarian tissues for IL-18, IL-18R, and IL-18BP. Immunohistochemical staining of normal ((a), (f), (k)) and different types of cancerous ovarian tissues: serous papillary ((b), (g), (l)), mucinous ((c), (h), (m)), and endometrioid ((d), (i), (n)) with mouse anti-human IL-18 ((a)–(d)), mouse anti-human IL-18R ((f)–(i)), and goat anti-human IL-18BP ((k)–(n)) antibodies. Both normal (j) and cancerous ((e), (o)) tissues were used as negative control. EP: epithelial cells and S: stroma. Magnification: ×600.

Both NOT and COT were stained positively when mouse anti-human IL-18 antibodies were applied. However, IL-18 expression could be detected mainly in epithelial cells, while stromal cells expressed low levels of IL-18 (Figures 3(a)–3(d)). NOT expressed high levels of IL-18R in both epithelial and stromal cells (Figure 3(f)). COT showed differences in the expression pattern of IL-18R. In the serous papillary carcinoma and mucinous carcinoma tissues, the cancerous epithelial cells expressed higher levels of IL-18R than the stroma cells (Figures 3(g) and 3(h), resp.). In contrast, endometrioid carcinoma expressed similar levels of IL-18R in both stromal and cancerous cells (Figure 3(i)). IL-18BP staining in NOT was high in the epithelial cell layer and weak in the stromal cells (Figure 3(k)). However, while in the serous papillary carcinoma and mucinous carcinoma tissues the main IL-18BP expression was by epithelial tumor cells (Figures 3(l) and 3(m), resp.), in the endometrioid carcinoma IL-18BP was expressed in similar levels in both the epithelial cells and the stroma cells (Figure 3(n)). Patterns of IL-18 and IL-18BP expression were similar in NOT and COT, namely, a dominant expression of both cytokines in the epithelial cells. In contrast, there were differences in the expression patterns of IL-18R in NOT compared to COT. While COT showed IL-18R expression that was similar to the IL-18 and IL-18BP expression patterns, NOT expressed similar levels of IL-18R in both stromal and epithelial cells.

## 4. Discussion

IL-18 is a multifunctional cytokine that has a critical role in ovarian physiologic function, inflammation, and immune response to cancer. It can either facilitate or impede tumor development and progression. Within the normal ovary it is involved in follicular development and atresia, ovulation, and steroidogenesis [12]. In this study, we have shown for the first time the expression levels and localization of IL-18BP and IL-18R in NOT and COT and cells. We have also shown higher levels of IL-18 in COT as compared to NOT. The ratio of IL-18/ IL-18PB, which indicates the actual echelon of IL-18 activity, was significantly higher in COT compared to NOT. Using immunohistochemical staining we have demonstrated that in both NOT and COT IL-18 and IL-18BP were predominantly expressed in epithelial cells. IL-18R was equally expressed in epithelial and stromal cells of NOT. However, in COT dominant epithelial staining for IL-18R was observed.

IL-18 has been shown to have procancerous effects [10–12]. Specifically, it can promote tumor growth and invasion by induction of Th-2 immune responses, inhibit recognition of cancer cells by antigen presenting cells, thus allowing the tumor to escape immune surveillance, increase vascular cell adhesion molecule expression, and augment the production of angiogenic factors that may promote a prometastatic microenvironment. IL-18 has also been implicated in anticancer activity which is mediated mainly through the stimulation of Th-1 immune response. It seems that IL-18 initially stimulates the nonspecific arm of the immune response through the activation of natural killer (NK) cells, and this is followed by the development of a specific cytotoxic T-cell-mediated antitumor response [20]. Furthermore, IL-18 encourages IFN-*γ*, IL-12, IL-2, TNF-*α*, IL-1*α*, and granulocyte macrophage-colony stimulating factor (GM-CSF) production but decreases IL-10 production [18].

The circulating levels of IL-18 are increased in patients with epithelial ovarian cancer compared to healthy controls and patients with borderline ovarian tumors and early-stage carcinoma [18, 19]. Although macrophages were considered the main source producer of IL-18, this cytokine is synthesized by many types of immune and nonimmune cells. Thus, it is not clear which cells synthesize IL-18 in different circumstances [21]. Since IL-18 mRNA and IL-18 protein were detected in primary ovarian cell cultures, it is reasonable to assume that the elevated IL-18 levels in ovarian cancer patient sera are produced by ovarian carcinoma cells as well as tumor-activated immune cells [19]. By using ELISA and semiquantitative RT-PCR techniques, we have shown that COT produced significantly higher levels of IL-18 and IL-18 mRNA compared to NOT. On the other hand, while NOT produced significantly higher levels of IL-18BP than COT, IL-18BP mRNA levels in NOT were almost similar to those in COT. Furthermore, IL-18R mRNA levels were similar in NOT and COT. In COT most of the receptors are in the epithelial cells, as depicted by immunohistochemical staining. These results may indicate that in carcinomatous epithelial ovarian cells the higher level of IL-18 is not counteracted by a similarly higher level of IL-18BP compared to normal epithelial ovarian cells. Nevertheless, the IL-18R mRNA levels are almost identical in COT and NOT. Therefore, it seems reasonable to assume that IL-18 is more potent/active in ovarian carcinoma cells.

Our findings implicate that the main source of IL-18 in the ovary is the epithelial cells; however, the target cells for IL-18 are different in normal and cancerous ovarian cells. It is reasonable to presume that, in mucinous and serous papillary ovarian carcinoma cells, IL-18 affects epithelial cells in an autocrine/paracrine manner. In normal ovarian cells and endometrioid type ovarian carcinoma cells, IL-18 binds to receptors in both stromal and epithelial cells. Thus, different regulatory mechanisms control the effect of IL-18 in the cell microenvironment of normal and cancerous ovarian cells. Our results indicate the significance of IL-18BP regulation mechanism. IL-18BP attenuates IL-18 effect in the cellular microenvironment. IL-18 activity, expressed as the ratio between IL-18 and IL-18BP levels, was significantly higher in cancerous ovarian cells compared to normal cells. Additional regulatory mechanisms for IL-18 have been described. Wang et al. [22] suggested that the ability to process IL-18 is lost during neoplastic transformation, particularly by downregulation of IL-18 or of IL-1*β*-converting enzyme (ICE) gene expression. Others [23, 24] emphasized the importance of two single nucleotide polymorphisms (SNPs) at positions −607 (C/A) and −137 (G/C) in the promoter region of the IL-18 gene. C allele at position −607 and the G allele at position −137 were attributed to the IL-18 higher transcription and protein production. Gaggero et al. [25] revealed the existence of an alternatively spliced variant of IL-18 mRNA, which lacked exon-3 (Δ3pro-IL-18). This variant cannot be cleaved by caspase-1 or caspase-4 to a biologically active form, but it retained the capacity to bind caspase-1, thus downregulating biologically active IL-18 production. IL-18BP prevents the binding of mature IL-18 to its receptor and inhibits IL-18 biological activity [26]. Therefore, the intracellular and local concentrations of IL-18 and IL-18BP determine the biological effects of IL-18 in physiological and pathophysiological context [12]. The majority of studies on serum IL-18 levels in cancer patients do not provide concomitant information about the concentration of its antagonist, that is, IL-18BP or IL-18-inducible cytokines such as IFN-*γ* in the same blood samples. Therefore, it would be important to know whether IL-18BP level is sufficient to counteract the pleiotropic effects of IL-18 in the different stages and clinical conditions of cancer patients [27]. Studies done to determine the prognostic potential of serum IL-18 levels in ovarian carcinoma patients failed to demonstrate a correlation with either stage or histology and it is clear that IL-18 alone cannot be used as a specific marker of ovarian cancer [19].

In conclusion, IL-18 is a novel cytokine with potent dual effects on tumor progression. Irrespective of its biological activity, IL-18 concentration significantly increases in the blood of ovarian carcinoma patients. Its production is a pathophysiologic feature of cancer connecting inflammatory and immune responses to cancer progression. However, the regulatory pathways for IL-18 production by both ovarian carcinoma cells and tumor-induced host cells and its mechanisms of action remain to be determined. To the best of our knowledge, our results are unique in delineating the action site of IL-18 in ovarian carcinoma. This is the first report to portray either qualitative or quantitative results concerning IL-18R and IL-18BP in primary COT. Additionally, we emphasize the significance of IL-18BP regulation mechanism in ovarian carcinoma progression. Nonetheless, our knowledge on the effects of IL-18 during human cancer development is still very limited and deserves further clinical investigation prior to considering IL-18 or IL-18BP to be potential therapeutic agents against cancer progression.

## Figures and Tables

**Table 1:** IL-18 and IL-18BP expression in normal and cancerous ovarian tissue homogenates.

Cell type	IL-18			IL-18BP		
	*N* (total)	*n* (positive)	Positive samples (%)	*N* (total)	*n* (positive)	Positive samples (%)
Normal	38	28	73.7	18	18	100
Carcinoma^a^	25	24	96	10	10	100
Serous papillary^b^	17	17	100	9	9	100
Endometrioid^b^	3	3	100	N.D	N.D	N.D
Undifferentiated^b^	3	2	66.7	N.D	N.D	N.D
Clear cell^b^	2	2	100	1	1	100

Normal and cancerous ovarian samples were homogenized and examined for IL-18 and IL-18BP protein levels by specific ELISA kits. The sum of the total samples tested (*N*), the sum of the positive expression samples (*n*), and the percentage of the positive samples of the total samples examined (%) are presented. N.D: not determined. ^a^Total cancerous samples; ^b^division into the different types of cancer.

**Table 2:** IL-18, IL-18BP, and IL-18R mRNA expression in normal and cancerous ovarian homogenates.

mRNA expression	Normal			Carcinoma		
	*N* (total)	*n* (positive)	Positive samples (%)	*N* (total)	*n* (positive)	Positive samples (%)
IL-18	16	13	81.25	15	10	66.67
IL-18BP	14	12	85.71	10	10	100
IL-18R	13	10	76.92	11	7	63.63

Normal and cancerous ovarian samples were homogenized and examined for IL-18, IL-18BP, and IL-18R mRNA levels by semiquantitative RT-PCR. The sum of the total samples tested (*N*), the sum of the positive expression samples (*n*), and the percentage of the positive samples of the total samples examined (%) are presented.
